# Hydrogen-Rich Water Suppresses Dark- and ABA-Induced Postharvest Senescence in Non-Heading Chinese Cabbage (*Brassica rapa* ssp. *chinensis*)

**DOI:** 10.3390/antiox15050554

**Published:** 2026-04-27

**Authors:** Yong Luo, Xinman Wang, Mengya Yin, Ranze Zhao, Dingyu Zhang, Hongfang Zhu

**Affiliations:** 1 Shanghai Key Laboratory of Protected Horticultural Technology, Horticultural Research Institute, Shanghai Academy of Agricultural Sciences, Shanghai 201403, China; ihenauly@163.com (Y.L.);; 2 Joint Center for Single Cell Biology, School of Agriculture and Biology, Shanghai Jiao Tong University, Shanghai 200240, China

**Keywords:** hydrogen-rich water, antioxidant, leaf senescence, abscisic acid, non-heading Chinese cabbage, ROS homeostasis

## Abstract

Non-heading Chinese cabbage (NHCC) is a highly economically valuable leafy vegetable widely grown in Asian regions. However, it undergoes rapid leaf yellowing and wilting during postharvest storage, which subsequently cause rapid quality decline and loss of nutritional components. Abscisic acid (ABA) promotes postharvest leaf senescence, while hydrogen-rich water (HRW) is widely used in postharvest preservation due to its excellent antioxidant properties; yet, the mechanism through which they interact to regulate postharvest senescence in NHCC remains unclear. Herein we found that exogenous HRW effectively delayed dark- and ABA-induced postharvest leaf senescence in NHCC, significantly maintained chlorophyll content, inhibited oxidative damage, and preserve nutritional components such as soluble sugars and vitamin C. The underlying mechanism was HRW inhibiting chlorophyll degradation by repressing the expression of chlorophyll catabolic genes like *NYC1*, *NYE1*, and *PPH1*. Meanwhile, HRW effectively lowered the accumulation of MDA and H_2_O_2_, elevated both the enzymatic activities and transcript abundance of *SOD* and *CAT*, and downregulated the transcript levels of *RbohB*, *RbohC*, *RbohD*, and *RbohE*, thereby maintaining reactive oxygen species (ROS) homeostasis. In addition, HRW negatively regulated ABA biosynthesis by inhibiting the transcript levels of *ABA1*, *ABA2* and *ABA3*, while promoting the transcription of *CYP707A1*, *CYP707A2* and *CYP707A3*. It also dampened the transcript abundance of ABA signaling components including *PYL5*, *ABI1*, and *ABF3*, thus blocking ABA signal transduction and alleviating its senescence-promoting effect. Collectively, this study confirms that HRW mitigates leaf senescence induced under dark and ABA conditions in NHCC via multiple synergistic pathways.

## 1. Introduction

As a typical Brassica crop, non-heading Chinese cabbage (NHCC, *Brassica rapa* ssp. *chinensis*) is economically important among the leafy vegetables, valued for its rich nutritional composition, delicate taste, and excellent environmental adaptability [1,2]. With major subspecies such as Pakchoi and Chinese flowering cabbage (CFC), it serves as a vital economic crop across Asia [3,4]. However, as a leafy vegetable, NHCC maintains vigorous metabolic activity after harvest, making it highly vulnerable to leaf yellowing, dehydration, nutrient depletion, and decay. Such rapid quality deterioration severely reduces its commercial value and causes economic losses [5,6]. Therefore, exploring the physiological and molecular mechanisms underlying leaf senescence offers a vital basis for establishing efficient postharvest preservation approaches aimed at extending shelf life and reducing economic losses [7,8,9].

Leaf senescence represents a highly programmed physiological process that is under the coordinated control of internal regulators such as plant age and phytohormones, along with external environmental stimuli. It is characterized by the orderly degradation and functional transition of cellular and subcellular structures and is critical for plant productivity and adaptability [10,11,12]. During early senescence, the ultrastructure of chloroplasts becomes disorganized as thylakoid proteins and stromal enzymes are progressively degraded, thereby triggering the programmed breakdown of chlorophyll [6,13]. Such catabolic progression is mediated by the elevated activities of a range of enzymes responsible for chlorophyll breakdown, among which are chlorophyll b reductase members NYC1 and NOL, together with PPH, PAO, RCCR, as well as NYE1 and NYE2 [10,14]. Consistent with these enzymatic changes, the transcript abundance of the corresponding CCE-encoding genes shows a gradual increase along with the progression of senescence [7,15]. Plant hormones, particularly abscisic acid (ABA) and ethylene, act as pivotal positive regulators of leaf senescence [10,16,17]. Endogenous ABA levels increase dramatically during leaf maturation and senescence, accompanied by the sustained activation of the pathways governing ABA production and signal transmission [12]. The ABA signaling cascade plays a central function in modulating the transcription profiles of genes responsible for chlorophyll breakdown and those associated with senescence. In *Arabidopsis*, ABA-responsive transcription factors ABF2/3/4 have been confirmed to positively promote the transcription of chlorophyll catabolic genes (*CCGs*, including *NYE1*, *NYC1*, *NYE2*, and *PAO*) and senescence-associated genes (*SAGs*, such as *SAG29* and *SAG12*), thereby promoting ABA-mediated leaf senescence [18]. In NHCC, studies revealed that melatonin delays postharvest leaf senescence by blocking ABF-regulated ABA biosynthesis and chlorophyll catabolism [7]. ABA-induced *NAC041* has been shown to repress the transcription of the ABA catabolic gene *CYP707A3*, resulting in ABA accumulation and accelerated leaf senescence [19]. Furthermore, ABA is capable of accelerating leaf senescence through elevating the acetylation levels of histones H3 and H4 on the promoter regions of *CCGs* [20]. Recent investigations have shown that diacetyl is able to retard postharvest leaf senescence via inhibiting *NAC100* transcription, consequently lowering the expression of its downstream targets *NCED3* and *ZEP*, thereby decreasing ABA accumulation [21]. Collectively, these findings uncover the crucial function of ABA and its regulatory network in modulating the postharvest senescence of this leafy vegetable, although the comprehensive regulatory network remains to be further elucidated.

As crucial signaling molecules, reactive oxygen species (ROS) participate in modulating diverse processes throughout plant growth, along with adaptive responses to external environmental cues [22]. However, excessive ROS accumulation triggers oxidative stress, which inflicts irreversible harm to cell membranes, protein structures and genomic DNA, ultimately speeding up the progression of leaf senescence [23]. In plant leaves, chloroplasts serve as the major sites for ROS generation, with disordered photosynthetic electron transport in senescing chloroplasts leading to massive ROS burst and release [24]. Consequently, the dynamic equilibrium of ROS homeostasis is as a key determinant triggering and accelerating leaf senescence [24,25]. To maintain ROS balance, plants have evolved sophisticated enzymatic systems for ROS generation and scavenging. Among these, respiratory burst oxidase homologs (RBOHs) comprise a pivotal enzyme family responsible for ROS production during biotic and abiotic stress responses [26]. RBOH family members act as core regulators that drive ROS production and thereby modulate plant growth, development and senescence [27,28]. The tight crosstalk among ROS, hormone signaling, and transcriptional regulation thus forms the complex and orderly regulatory network governing leaf senescence.

Hydrogen-rich water (HRW) is an aqueous solution containing dissolved molecular hydrogen (H_2_) and has emerged as a promising agent for hydrogen application in biological systems owing to its non-toxicity, good water solubility, and potent ability to selectively scavenge toxic free radicals via mild reduction [29,30]. The significant postharvest preservation benefits of HRW have been demonstrated in various horticultural crops, including in kiwifruit, okra, and broccoli. In kiwifruit, HRW treatment delays ripening by reducing respiration rate, enhancing SOD activity, and alleviating lipid peroxidation [31]. In okra, HRW retards postharvest senescence by modulating the crosstalk among multiple phytohormones, including increasing GA, IAA, and melatonin contents while decreasing ABA levels [32]. In broccoli, HRW delays postharvest yellowing by inhibiting the increase in ABA content during storage [33]. The underlying mechanisms primarily involve the regulation of ROS metabolism, enhancement of the antioxidant machinery, preservation of cellular integrity and energy status, as well as modulation of hormone crosstalk. Despite the well-demonstrated potential of HRW in delaying postharvest senescence, its efficacy and associated molecular mechanisms in NHCC, especially the crosstalk with ABA signaling, remain poorly understood.

With the aim of exploring the effects and molecular mechanism underlying the HRW-mediated regulation of postharvest senescence in NHCC under dark and ABA induction, we integrated phenotypic observation, physiological measurement, molecular detection and transcriptomic analysis. Our results demonstrate that HRW delays leaf senescence and maintains nutritional quality by inhibiting ABA biosynthesis and signaling, suppressing chlorophyll degradation, and enhancing antioxidant capacity. These results establish a theoretical foundation for HRW-based postharvest storage strategies of leafy vegetables and deepen our understanding of hydrogen-mediated regulation in plant senescence.

## 2. Materials and Methods

### 2.1. Plant Materials and Culture Environment

Selected uniform seeds of the NHCC cultivar Pakchoi (*Brassica rapa* ssp. *chinensis*) were initially sown and germinated on moistened filter paper. Subsequently, each seedling was transplanted into a separate plastic pot filled with a 3:1 (*v*/*v*) mixture of peat soil and perlite. Following transplantation, the seedlings were grown in a climate-controlled growth chamber under a 16 h light/8 h dark cycle, at a photosynthetic photon flux density of roughly 300 μmol·m^−2^·s^−1^, 22 °C, and a relative humidity of 60%. At 35 days of cultivation, the plants were harvested for further experimental analyses.

### 2.2. Chemical Treatments

The experiment comprised five groups: (1) Pre: untreated samples, collected immediately after harvest; (2) Water: distilled water treatment; (3) HRW: 25% HRW treatment, and HRW was prepared using a TH-300 hydrogen-producing apparatus (Beijing Zhonghuipu Analytical Technology Co., Ltd., Beijing, China). High-purity hydrogen (99.99%, *v*/*v*) was bubbled continuously into 8 L of deionized water at 25 °C at a flow velocity of 300 mL·min^−1^ for 160 min to obtain saturated HRW. To prepare 25% HRW, the saturated solution was diluted with pure water in a volume ratio of 1:3 (saturated HRW: pure water, *v*/*v*). (4) ABA: abscisic acid treatment (50 μM). ABA (plant hormone grade, purity ≥ 98.0%) was obtained from Sangon Biotech Co., Ltd. (Shanghai, China). (5) ABA + HRW: co-treatment of 50 μM ABA and 25% HRW. Each treatment was performed in triplicate biologically. After harvest, plant materials were placed in plastic boxes (36 × 21 × 13 cm) lined with filter paper, with three plants per box. A total of 150 mL of the corresponding treatment solution was added to each box, and the samples were stored at 22 °C in darkness for 3 days. Phenotypic changes were recorded, and samples were collected for subsequent experiments.

### 2.3. Measurement of Chlorophyll Content

A SPAD-502Plus chlorophyll meter (Konica Minolta, Tokyo, Japan) was used to determine the relative chlorophyll content [34]. For each treatment, three representative leaves were selected, and three independent tests were performed on distinct regions of every leaf to ensure data accuracy and reproducibility.

### 2.4. Chlorophyll Fluorescence Imaging and Fv/Fm Analysis

The chlorophyll fluorescence parameter Fv/Fm (maximal PSII photochemical efficiency) was characterized and visualized by a pulse-amplitude-modulated imaging system (IMAGING-PAM-MAX/L, Walz, Effeltrich, Germany). Before relevant determinations, leaf samples were subjected to dark adaptation for 20 min to guarantee the full relaxation of the photosynthetic reaction centers.

### 2.5. Measurement of Relative Ion Leakage

Leaf membrane integrity was evaluated by measuring the relative ion leakage according to an established protocol [35]. Briefly, leaf samples were fully submerged in centrifuge tubes containing deionized water and kept at 25 °C for 2 h. The initial electrical conductivity (T1) of the bathing solution was measured using a digital conductivity meter (Waterproof ECTestr11+ Multi-Range Tester, Eutech Instruments, Singapore). Subsequently, the samples were boiled for 30 min to achieve complete electrolyte release. After being cooled to room temperature, we measured the final electrolyte conductivity (T2). Relative ion leakage was computed according to Equation (1) given below:

(1) (T1/T2) × 100%

### 2.6. Histochemical Detection of ROS

To observe the in situ accumulation of ROS, histochemical staining was carried out by employing 3,3′-diaminobenzidine (DAB, biochemical grade, purity ≥ 96.0%) and nitroblue tetrazolium (NBT, biochemical grade, purity ≥ 98.0%), both obtained from Sangon Biotech (Shanghai, China). Leaf samples were kept in DAB solution (1 mg·mL^−1^, pH 3.8) for the detection of hydrogen peroxide (H_2_O_2_), while NBT solution (0.5 mg·mL^−1^, pH 7.8) was used to visualize superoxide anions (O_2_^−^). Following dark incubation for 6 h, leaf samples were placed into 95% (*v*/*v*) ethanol and heated in a boiling water bath until complete bleaching of chlorophyll. The decolorized samples were stored in 95% ethanol and photographed using a camera.

### 2.7. Determination of Quality Index

According to previous reports, the 3,5-dinitrosalicylic acid (DNS) method was used to determine the content of reducing sugars [36]. In brief, 0.1 g of fresh leaf was homogenized, followed by extraction with deionized water. The resulting supernatant was mixed with DNS reagent, followed by incubation in a boiling water bath and absorbance measurement at 540 nm. The total phenolic content of leaf samples was measured using the Folin–Ciocalteu assay [37]. In short, after grinding 0.1 g of fresh leaf, extraction was performed using ethanol. The collected supernatant was combined with Folin–Ciocalteu reagent and sodium carbonate solution, followed by spectrophotometric detection at 765 nm. The flavonoid content was quantified according to a reported protocol [38]. After homogenizing 0.1 g of fresh leaf with ethanol, the supernatant was combined with aluminum chloride and sodium nitrite solution, and absorbance was monitored at 510 nm.

The contents of soluble sugars, vitamin C, and soluble proteins were determined using corresponding commercial kits (catalogue numbers KT-1-Y, ASA-1-W, and KMSP-1-W, respectively; Suzhou Comin Biotechnology Co., Ltd., Suzhou, China) following the vendor’s protocols.

### 2.8. qRT-PCR Analysis

RNA was extracted from leaves of NHCC with an RNA purification kit (Vazyme, Nanjing, China), following the recommended protocol. Subsequently, RNA was reverse-transcribed into cDNA using a HiScript II Q RT SuperMix kit (Vazyme, catalog no. R223-01). qRT-PCR analysis was subsequently performed according to our previously protocol [34]. The 2^−ΔΔCt^ approach was used to quantify gene expression levels. Primer sequences are listed in Supplementary Table S1.

### 2.9. Transcriptome Sequencing and Analysis

RNA was extracted from leaves, followed by library construction, quality control, and sequencing on an Illumina NovaSeq 6000 platform (Illumina, San Diego, CA, USA). All procedures were performed by Smartgenomics Technology Institute (Qingdao, China). Raw sequencing reads were preprocessed for acquiring high-quality clean reads and subsequently mapped to the Brassica rapa NHCC001 reference genome employing the HISAT2 aligner [1]. Gene transcription levels were calculated, differentially expressed genes (DEGs) were screened, and GO and KEGG enrichment analyses were performed with reference to our recent research protocol [34].

### 2.10. Statistical Analysis

Statistical analyses were performed using SPSS 25.0 (IBM, Armonk, New York, USA). Data are shown as mean ± SD based on three independent biological replicates. Student’s *t*-test was used to analyze differences between two groups, and one-way ANOVA followed by LSD test was applied for comparisons among multiple groups.

## 3. Results

### 3.1. Exogenous HRW Delays Leaf Senescence Induced by Dark and ABA in Postharvest NHCC

**Figure 1:**
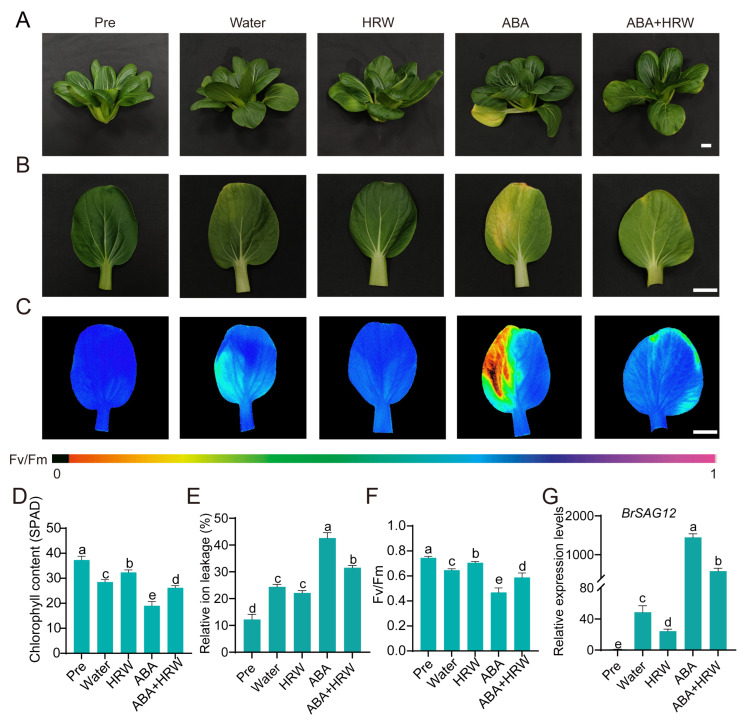
Exogenous HRW delays dark- and ABA-induced postharvest leaf senescence in NHCC. (**A**) Phenotype of NHCC and (**B**) detached leaves following 3 d of dark treatment at 22 °C. (**C**) Chlorophyll fluorescence images showing the maximal PSII photochemical efficiency (Fv/Fm). (**D**) Relative chlorophyll content (SPAD). (**E**) Relative ion leakage. (**F**) Quantitative analysis of Fv/Fm. (**G**) Relative expression levels of *BrSAG12*. Bar = 2 cm. Data are means ± SD. Distinct letters denote significant differences (*n* = 3, *p* < 0.05, one-way ANOVA).

To investigate whether exogenous HRW alleviates the senescence of harvested NHCC and whether this process involves the ABA pathway, phenotypic, physiological, and molecular indices were examined under different treatments. The results showed that after 3 d of dark storage at 22 °C, water-treated leaves exhibited obvious yellowing, whereas HRW-treated leaves retained a greener appearance. ABA treatment markedly accelerated leaf yellowing, while co-treatment with HRW significantly alleviated ABA-induced leaf yellowing (Figure 1A). Compared with the water treatment, the HRW treatment significantly alleviated the decline in the chlorophyll content and Fv/Fm, reduced the relative ion leakage, and suppressed the expression of the senescence marker gene *BrSAG12*. ABA treatment exerted opposite effects to those of HRW on these indices. All measured parameters in the HRW+ABA co-treatment group were between those of the water control and ABA alone treatment (Figure 1B–G). Exogenous HRW effectively delays postharvest leaf senescence in NHCC, and this effect is mediated, at least in part, by antagonizing the ABA signaling pathway.

### 3.2. HRW Delays Dark- and ABA-Induced Leaf Senescence Through Suppressing Expression Levels of Chlorophyll-Degradation-Related Genes

**Figure 2:**
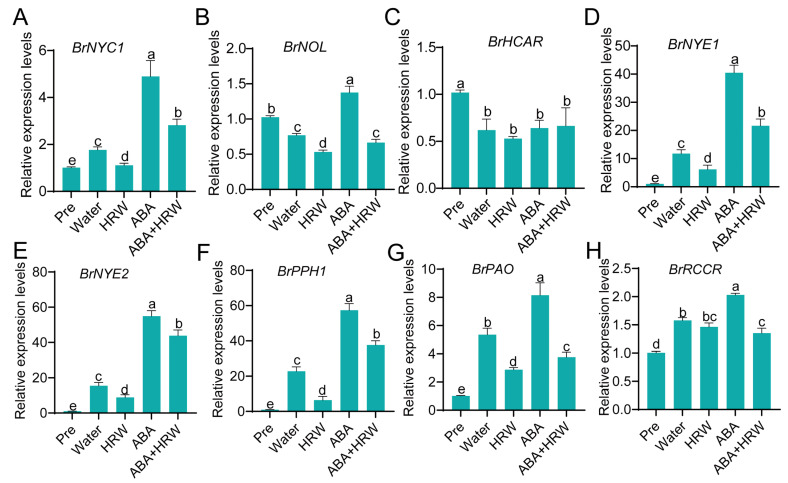
Exogenous HRW suppresses expression of key *CCGs* under dark and ABA conditions in postharvest NHCC. (**A**–**H**) Relative expression levels of *CCGs* in postharvest NHCC leaves under different treatment conditions: (**A**) *BrNYC1*, (**B**) *BrNOL*, (**C**) *BrHCAR*, (**D**) *BrNYE1*, (**E**) *BrNYE2*, (**F**) *BrPPH1*, (**G**) *BrPAO*, (**H**) *BrRCCR*. Data are means ± SD. Distinct letters denote significant differences (*n* = 3, *p* < 0.05, one-way ANOVA).

Leaf yellowing represents the most striking symptom of senescence, characterized at the biochemical level by the systematic activation of chlorophyll catabolism [14]. To uncover the molecular basis underlying the HRW-mediated inhibition of dark- and ABA-triggered leaf yellowing, we examined the expression levels of the major genes participating in chlorophyll degradation. The qRT-PCR results revealed that, compared with the Pre (pre-treatment, fresh harvest) group, most *CCGs* were significantly upregulated in the water control, except for *BrNOL* and *BrHCAR*. Notably, this induction was markedly suppressed by HRW treatment, suggesting that HRW maintains chlorophyll content by systematically repressing the transcription of key chlorophyll degradation genes. ABA treatment strongly activated the expression of *BrNYC1*, *BrNYE1*, *BrNYE2*, *BrPPH1*, and *BrPAO*. However, such ABA-mediated induction was significantly suppressed by HRW co-treatment (Figure 2). These molecular results are highly consistent with the leaf phenotypic observations. Our results demonstrate that HRW delays dark- and ABA-induced leaf yellowing and senescence in postharvest NHCC by repressing key *CCGs* and ABA-promoted chlorophyll degradation.

### 3.3. HRW Alleviates Dark- and ABA-Induced Oxidative Damage and Enhances Antioxidant Defense in Postharvest NHCC

**Figure 3:**
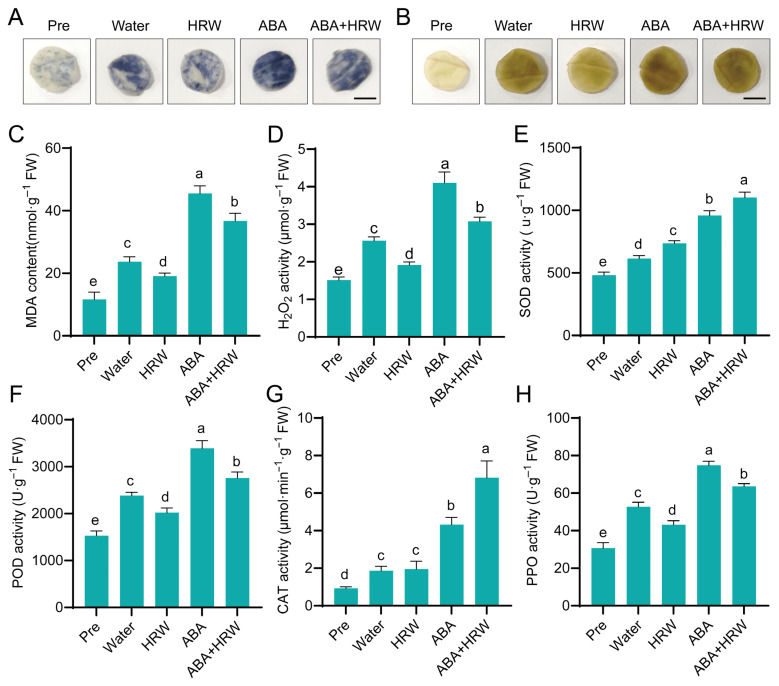
Exogenous HRW modulates ROS accumulation and antioxidant capacity under dark and ABA conditions in postharvest NHCC. (**A**) Histochemical localization of superoxide anions (O_2_^−^) per NBT staining. (**B**) Histochemical localization of hydrogen peroxide (H_2_O_2_) per DAB staining. (**C**) MDA content. (**D**) H_2_O_2_ content. (**E**–**H**) Activities of antioxidant-related enzymes: (**E**) superoxide dismutase (SOD), (**F**) peroxidase (POD), (**G**) catalase (CAT), (**H**) polyphenol oxidase (PPO). Bar = 0.5 cm. Data are means ± SD. Distinct letters denote significant differences (*n* = 3, *p* < 0.05, one-way ANOVA).

As a redox signaling molecule in plants, molecular hydrogen (H_2_) exerts vital functions in preserving cellular redox balance and antioxidant defenses [39]. To elucidate the regulatory effect of HRW on ROS metabolism in leaf senescence, we systematically analyzed ROS accumulation, oxidative damage levels, and antioxidant defense indices. Histochemical staining revealed that the leaves in the water control group exhibited obvious dark blue spots following NBT staining for O_2_^−^ as well as brown precipitates following DAB staining for H_2_O_2_, indicating severe ROS accumulation during postharvest storage. Compared with the water control, exogenous ABA treatment further intensified these staining signals, suggesting that ABA aggravates ROS overproduction. In contrast, HRW treatment apparently reduced the staining intensity, indicating that HRW inhibits the excessive accumulation of H_2_O_2_ and O_2_^−^. Notably, the ABA-induced increase in staining was significantly alleviated by HRW and ABA co-treatment (ABA + HRW) (Figure 3A,B). Consistent with the staining results, quantitative measurements confirmed that HRW significantly reduced the H_2_O_2_ and MDA contents, whereas ABA had the opposite effect (Figure 3C,D).

Given the crucial roles of antioxidant enzymes in modulating H_2_O_2_ and MDA levels, we examined the activities of key antioxidant enzymes. The results showed that compared with the pre-treatment (Pre) group, the activities of SOD, POD, CAT, and PPO were significantly elevated in all treatment groups. The POD and PPO activities were highest under ABA treatment, while ABA + HRW treatment inhibited these two enzymes but most strongly induced SOD and CAT activities (Figure 3E–H). These results demonstrate that stress conditions during postharvest storage induce an increase in antioxidant enzyme activities. HRW maintains redox homeostasis by differentially regulating antioxidant enzyme activities, which not only reduces the excessive activation of POD and PPO induced by ABA but also strongly enhances SOD and CAT activities.

### 3.4. HRW Modulates Antioxidant and ROS-Production Genes to Alleviate Dark- and ABA-Induced Oxidative Stress at Transcriptional Level

**Figure 4:**
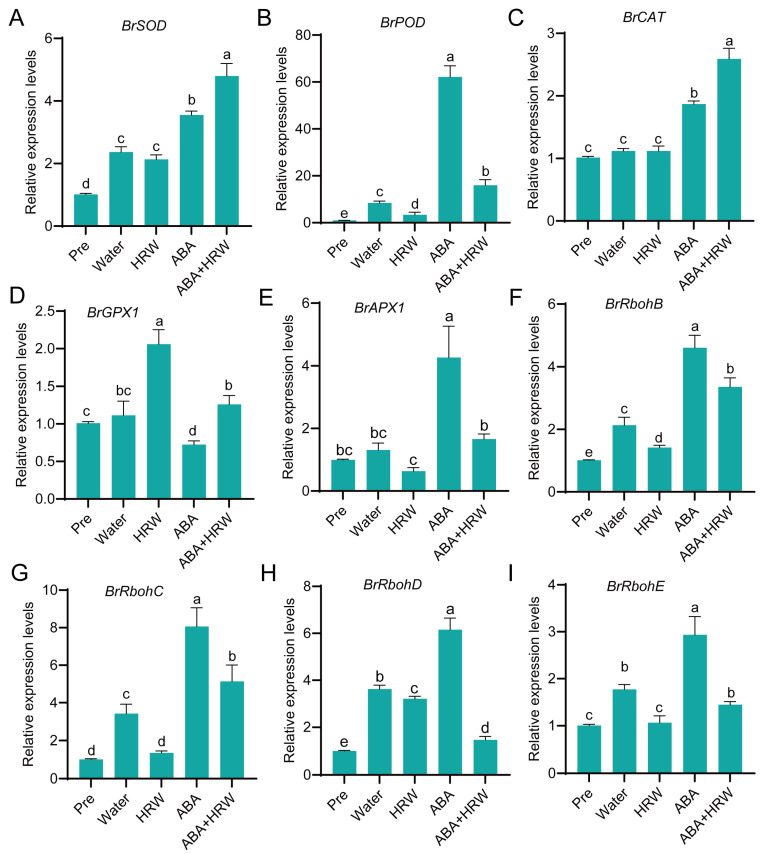
HRW represses dark- and ABA-triggered ROS biosynthesis and regulates antioxidant-related gene expression. (**A**–**I**) Relative expression levels of antioxidant and ROS-producing genes in NHCC leaves under different treatments: (**A**) *BrSOD*, (**B**) *BrPOD*, (**C**) *BrCAT*, (**D**) *BrGPX1*, (**E**) *BrAPX1*, (**F**) *BrRbohB*, (**G**) *BrRbohC*, (**H**) *BrRbohD*, (**I**) *BrRbohE*. Data are means ± SD. Distinct letters denote significant differences (*n* = 3, *p* < 0.05, one-way ANOVA).

To further explore the molecular mechanism responsible for the altered antioxidant enzyme activities described above, we analyzed the transcript levels of antioxidant and ROS production genes. Different from the changes in enzyme activities, only *BrSOD* and *BrPOD* were significantly upregulated at the transcriptional level in all treatment groups compared with the Pre group. ABA treatment most strongly induced the expression of *BrPOD* and *BrAPX1*. ABA + HRW treatment led to the most significant increases in *BrSOD* and *BrCAT* expression, a finding in line with the corresponding enzymatic activities. Compared with the water control, HRW markedly enhanced the expression of *BrGPX1* (Figure 4A–E).

For ROS production genes (Rboh), ABA treatment caused the most pronounced induction. The transcript levels of *BrRbohB* corresponded with the severity of leaf senescence. Notably, ABA + HRW treatment significantly suppressed the ABA-induced upregulation of these Rboh genes, especially *BrRbohD* and *BrRbohE* (Figure 4F–I). Collectively, these results indicate that HRW maintains redox homeostasis at the transcriptional level by differentially regulating antioxidant genes and suppressing ROS-generating Rboh genes, thereby antagonizing dark- and ABA-triggered oxidative stress and leaf senescence.

### 3.5. Effects of HRW on Postharvest Quality of NHCC

**Figure 5:**
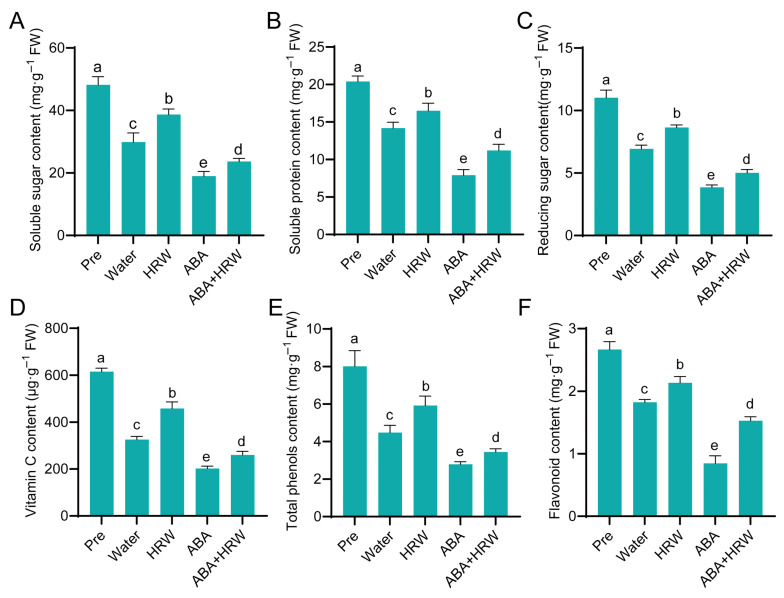
HRW maintains quality of postharvest NHCC leaves. (**A**–**F**) Contents of quality indices: (**A**) soluble sugar, (**B**) soluble protein, (**C**) reducing sugar, (**D**) vitamin C, (**E**) total phenols, (**F**) flavonoid. Data are means ± SD. Distinct letters denote significant differences (*n* = 3, *p* < 0.05, one-way ANOVA).

To assess the influence of HRW on the nutritional and edible traits of postharvest NHCC, we measured the contents of soluble sugar, soluble protein, reducing sugar, vitamin C, total phenolics, and flavonoids in leaves across various treatments. Compared with the Pre group, all treatments led to significant reductions in these indicators, indicating that dark-induced senescence caused a general decline in nutritional quality (Figure 5). Among these, the ABA treatment group exhibited the most severe loss of quality components, with the lowest levels of these indicators, suggesting that ABA accelerates senescence and quality deterioration. Notably, HRW treatment effectively mitigated this quality loss. Compared with the water group, HRW-treated leaves maintained significantly higher contents of these indicators. Furthermore, the ABA+HRW treatment group exhibited a clear reversal of the ABA-induced quality decline, demonstrating that HRW alleviates ABA-accelerated senescence and effectively preserves the postharvest quality of NHCC. These results indicate that HRW maintains the nutritional quality of postharvest NHCC by preserving key functional components including sugars, proteins, vitamin C, and antioxidant phenolics, thereby delaying senescence and extending the shelf life of the vegetable.

### 3.6. Transcriptomic Profiling Reveals HRW Delays Leaf Senescence by Inhibiting ABA Signaling and Senescence-Related Pathways

**Figure 6:**
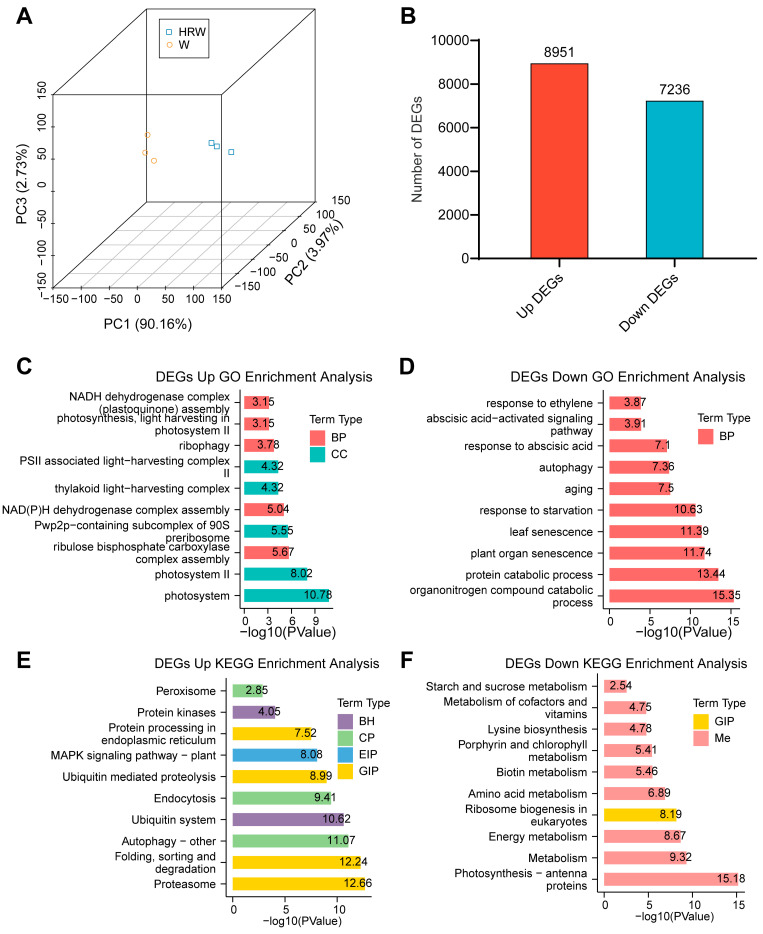
Comparative global transcriptomic analysis of gene expression in NHCC leaves in response to HRW treatment. (**A**) PCA analysis. (**B**) Statistical distribution of number of DEGs. (**C**,**D**) GO enrichment analysis. (**E**,**F**) KEGG pathway enrichment analysis.

To elucidate the global regulatory mechanism through which HRW delays leaf senescence, we conducted a comparative transcriptomic profiling between the control and HRW-treated samples. Principal component analysis (PCA) showed that samples from each treatment were closely clustered and clearly separated (Figure 6A). This marked separation indicated that HRW triggered extensive and significant transcriptional reprogramming in NHCC leaves during leaf senescence. In total, we identified 16,187 DEGs, including 8951 upregulated and 7236 downregulated genes in the HRW treatment relative to the water control (Figure 6B).

GO enrichment analysis showed that the upregulated DEGs were largely related to photosynthesis-related biological processes and cellular components, including NADH dehydrogenase complex, thylakoid structure, photosystem I and II, and photosynthetic electron transport (Figure 6C). In contrast, the downregulated DEGs displayed prominent enrichment in pathways linked to phytohormone responses and leaf senescence, such as ABA response, ABA-mediated signaling pathway, autophagy, ethylene response, leaf senescence, and protein degradation (Figure 6D). KEGG pathway enrichment analysis further confirmed these functional characteristics. The upregulated DEGs were mostly enriched in photosynthesis, proteasome pathway, phenylpropanoid biosynthesis, and plant MAPK signaling pathway (Figure 6E). The repressed DEGs were mainly related to light-harvesting antenna proteins and metabolisms of energy, porphyrin/chlorophyll, starch and sucrose (Figure 6F). Together, these transcriptomic results indicate that HRW delays postharvest leaf senescence at the global transcriptional level by maintaining the expression of photosynthesis and energy metabolism genes, while inhibiting the activation of senescence- and ABA-signaling–related pathways.

### 3.7. HRW Inhibits ABA Biosynthesis and Signaling to Alleviate Leaf Senescence

**Figure 7:**
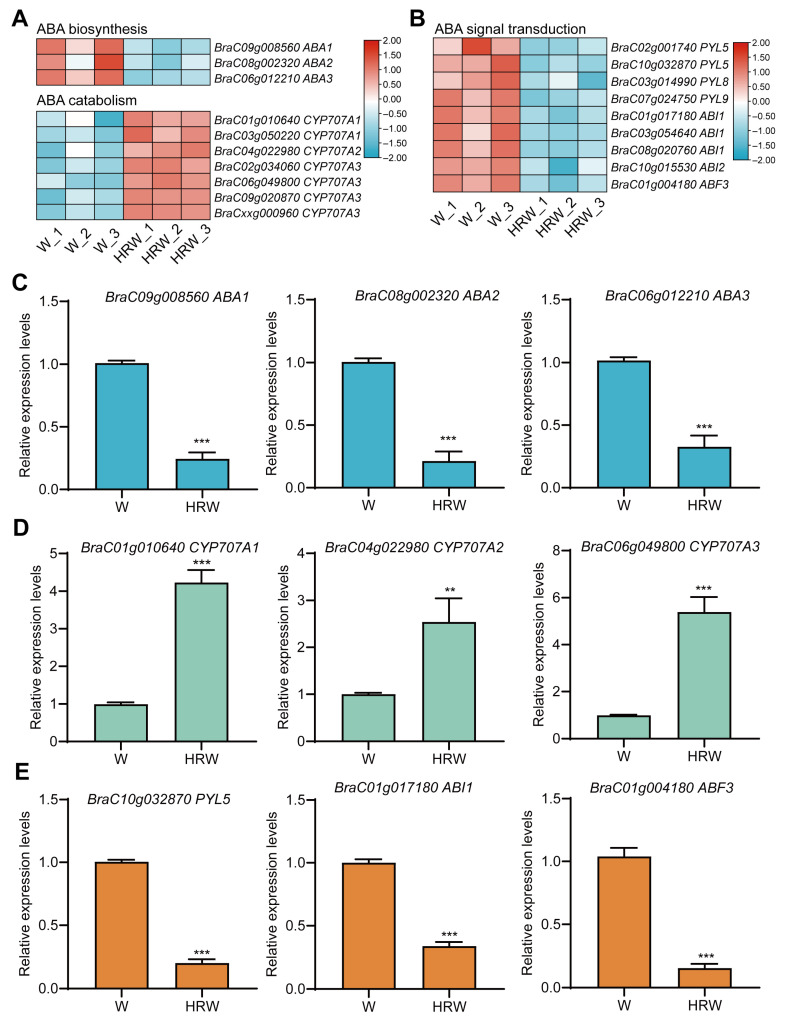
Heatmap and qRT-PCR verification of ABA-metabolism- and signaling-related genes. (**A**) Expression heatmap of ABA biosynthesis and catabolism genes. (**B**) Heatmap showing the transcriptional changes of key components in the ABA signal transduction pathway. (**C**–**E**) qRT-PCR validation: (**C**) ABA biosynthetic genes *BrABA1*, *BrABA2*, and *BrABA3*, (**D**) ABA catabolic genes *BrCYP707A1*, *BrCYP707A2*, and *BrCYP707A3*, (**E**) ABA signaling genes *BrPYL5*, *BrABI1*, and *BrABF3*. Data are means ± SD. Distinct letters denote significant differences (*n* = 3, ** *p* < 0.01, *** *p* < 0.001, *t*-test).

The ABA biosynthesis and response pathways being markedly suppressed at the transcriptional level suggested that HRW treatment inhibited dark-induced leaf senescence by suppressing ABA signaling, which was consistent with our earlier phenotypic observations. We therefore further screened DEGs involved in ABA biosynthesis, catabolism, and signal transduction. HRW treatment markedly downregulated the expression of ABA biosynthetic genes *BrABA1*, *BrABA2*, and *BrABA3*, while upregulating ABA catabolic genes *BrCYP707A1*, *BrCYP707A2*, and *BrCYP707A3* (Figure 7A). In addition, HRW extensively regulated the ABA signaling cascade. Specifically, relative to the water control, HRW significantly reduced the transcript of ABA receptor genes *BrPYL5*, *BrPYL8*, and *BrPYL9*, negative regulatory phosphatase genes *BrABI1* and *BrABI2*, and the downstream transcription factor gene *BrABF3* (Figure 7B).

We further verified the expression of several genes from the transcriptome data by qRT-PCR, and the results were consistent with the sequencing data. HRW treatment significantly inhibited the transcript levels of *BrABA1*, *BrABA2*, *BrABA3*, *BrPYL5*, *BrABI1*, and *BrABF3* but significantly promoted the expression of *BrCYP707A1*, *BrCYP707A2*, and *BrCYP707A3* (Figure 7C–E). Furthermore, we measured the endogenous ABA content in the leaves under different treatments. Compared with the Pre group, all four treatments significantly promoted ABA accumulation, with the most pronounced increase observed in the ABA treatment. Notably, ABA+HRW treatment markedly reduced ABA content relative to ABA treatment alone. Similarly, HRW treatment alone also decreased ABA accumulation compared with the water control (Figure S1). Collectively, these findings demonstrate that HRW effectively delays dark- and ABA-induced leaf senescence through comprehensively disrupting ABA accumulation and signaling transduction.

## 4. Discussion

Leaf yellowing constitutes the most typical characteristic of postharvest senescence in leafy vegetables, which is primarily attributed to the activation of chlorophyll catabolic pathways [3,5,14]. A set of core *CCGs*, including *NYC1*, *NOL*, *NYE1*, *PPH* and *PAO*, encode rate-limiting enzymes in chlorophyll breakdown, and their transcriptional upregulation is widely regarded as a molecular switch for leaf yellowing during senescence [14]. ABA has been well-established to induce the expression of these *CCGs* and thereby accelerate chlorophyll degradation in various horticultural crops [7,40]. Hydrogen is increasingly widely used in plants and has been applied for the postharvest preservation of fruits and vegetables [32,41]. The combination of HRW with pre-cooling treatment enhances antioxidant capacity and delays senescence in postharvest Pakchoi [42]. Pre-treatment with HRW also improves ROS-scavenging capacity and reduces decay in kiwifruit and mushrooms [31,43]. Further studies revealed that HRW delays the senescence of okra fruit mainly by increasing melatonin content and modulates cell wall metabolism and composition, thereby inhibiting the softening process [32,44]. In the present study, HRW treatment effectively alleviated dark- and ABA-induced leaf senescence and yellowing in NHCC (Figure 1). Consistent with phenotypic observations, dark storage significantly induced the transcript levels of major CCGs, and this induction was further aggravated by exogenous ABA application. Conversely, HRW treatment strongly repressed the transcription of key *CCGs,* including *BrNYC1*, *BrNYE1*, *BrPPH1* and *BrPAO*, and reduced the ABA-mediated upregulation of these genes (Figure 2). These results clearly demonstrate that HRW delays chlorophyll degradation mainly through the transcriptional repression of core *CCGs*. The transcriptome data further indicated that HRW maintained the expression of genes associated with chloroplast integrity, thylakoid structure, photosystems, and light-harvesting antenna proteins (Figure 6C,E). Given the essential role of chloroplast structural stability in sustaining chlorophyll retention and photosynthetic function [13,14], it is proposed that HRW delays leaf yellowing by both inhibiting chlorophyll degradation and preserving the structural and functional integrity of chloroplasts. A previous study reported that sucrose delayed chlorophyll breakdown by stabilizing the chloroplast ultrastructure in postharvest NHCC [6], suggesting that HRW employs a partially conserved mechanism in protecting chloroplast function. Collectively, these results indicate that HRW suppresses chlorophyll catabolism at the transcriptional level and sustains photosynthetic apparatus stability, thus contributing to higher chlorophyll content and Fv/Fm during postharvest storage.

ROS homeostasis acts as a central regulatory node in leaf senescence progression, and excessive ROS accumulation causes oxidative damage to cellular components, ultimately accelerating senescence syndrome [45,46]. To counteract oxidative stress, plants have evolved a sophisticated antioxidant system composed of enzymatic antioxidants such as SOD, POD and CAT, as well as Rboh family genes that mediate ROS generation [47,48]. Mounting evidence has demonstrated that HRW confers stress tolerance by reducing ROS overaccumulation and enhancing antioxidant capacity in diverse plant systems [30,31]. HRW significantly decreased the accumulation of H_2_O_2_, O_2_^−^ and MDA in NHCC leaves under dark and ABA stress (Figure 3A–D). Notably, ABA treatment led to elevated activities of POD and PPO, whereas HRW co-treatment mitigated these increases while markedly enhancing SOD and CAT activities (Figure 3E–H). This selective regulatory pattern implies that HRW does not indiscriminately activate all antioxidant enzymes, but orchestrates the antioxidant system in a precise and stress-adaptive manner. Furthermore, HRW upregulated the expression of *BrSOD*, *BrCAT* and *BrGPX1* while significantly inhibiting the ABA-induced expression of ROS-producing Rboh genes (Figure 4). These findings indicate that HRW delays leaf senescence, at least in part, via a dual molecular mechanism: promoting antioxidant gene expression to strengthen detoxification and repressing Rboh-mediated ROS production to restrain oxidative burst, which represents a key mechanistic basis for HRW-delayed leaf senescence.

Leaf senescence is a highly programmed physiological process governed by complex transcriptional networks, among which ABA functions as a critical senescence-promoting hormone that induces *SAGs* and accelerates senescence under dark or exogenous ABA treatment [10,12,18]. Our transcriptome analysis revealed that HRW induced widespread transcriptional reprogramming in harvested NHCC leaves, with numerous DEGs involved in various biological processes, which were highly enriched in pathways involved in plant hormone signal transduction, primary and secondary metabolism, and stress responses (Figure 6). These coordinated transcriptional changes collectively contribute to delayed senescence, reduced leaf yellowing, and improved nutritional quality including higher contents of soluble sugars, soluble protein, vitamin C and flavonoids (Figure 5), suggesting that the protective effects of HRW rely on the synergistic regulation of multiple biological pathways rather than a single target.

**Figure 8:**
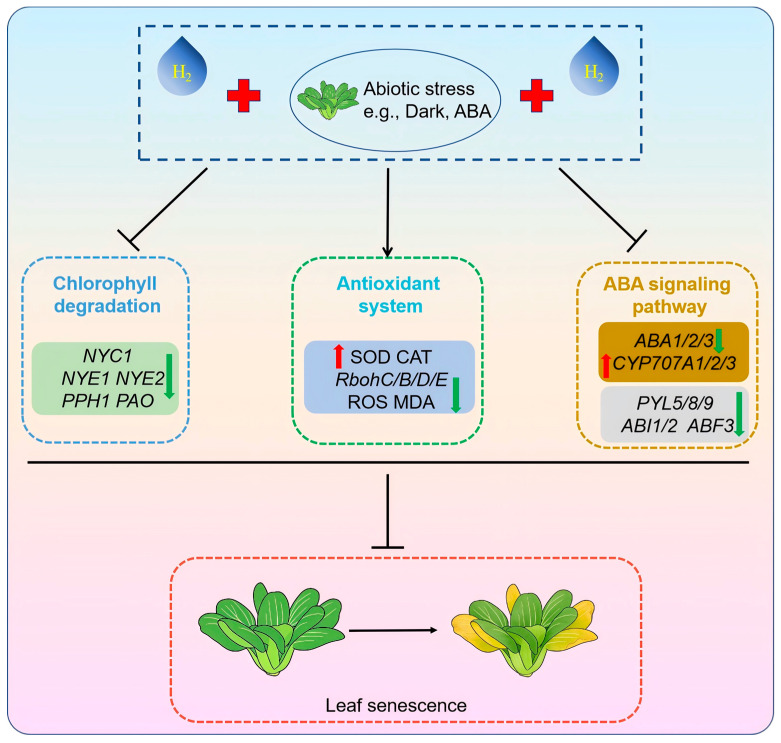
An integrated working model illustrating the mechanistic role of exogenous HRW in delaying postharvest leaf senescence in NHCC. HRW delays postharvest senescence through inhibiting chlorophyll degradation, fine-tuning ROS homeostasis to maintain cellular integrity, and systemically suppressing ABA metabolism and signal transduction.

ABA promotes leaf senescence primarily by activating *CCGs* and *SAGs*, thereby accelerating chlorophyll degradation and leaf yellowing [7,49,50]. In the present study, HRW significantly downregulated the expression of ABA biosynthesis and signaling pathway genes while upregulating the expression of ABA catabolic genes, thereby reducing endogenous ABA accumulation under dark and ABA stress (Figure 7 and Figure S1). Although the exact responsive genes may vary across plant species, HRW appears to employ a conserved regulatory strategy in modulating ABA homeostasis via the coordinated control of ABA biosynthesis, catabolism and signaling. Consistently, HRW has been documented to delay postharvest senescence in okra and broccoli by inhibiting ABA action [32,33]. Furthermore, our supplementary experiments on postharvest CFC confirmed that HRW similarly inhibited dark- and ABA-induced leaf senescence (Figures S2 and S3), supporting the conserved role of HRW in delaying leaf senescence in leafy vegetables. Based on the above results, we propose an integrated working model for the HRW-mediated delay of postharvest leaf senescence in NHCC (Figure 8). HRW exerts its protective role through three coordinated regulatory modules: it represses key chlorophyll catabolic genes to slow chlorophyll breakdown, maintains ROS homeostasis by enhancing antioxidant systems while suppressing ROS production, and antagonizes ABA-mediated senescence signaling via modulating ABA biosynthesis, catabolism and transduction.

## 5. Conclusions

Our study systematically reveals that HRW efficiently delays postharvest leaf senescence in NHCC via orchestrating multiple coordinated physiological and molecular pathways. Such regulation acts synergistically to maintain chloroplast structure and chlorophyll content, preserve nutritional quality, alleviate leaf yellowing, and thereby extend the postharvest shelf life. These results provide a novel understanding of the molecular basis of hydrogen-mediated senescence delay and highlight the practical value of HRW as a safe and effective approach for preserving leafy vegetables. Further research is warranted to explore the critical upstream transcription factors modulated by HRW in the ABA biosynthesis and signaling cascade. Moreover, clarifying the crosstalk between ABA and other phytohormones will further advance our understanding of the molecular network underlying HRW-delayed senescence.

## Data Availability

All datasets generated or analyzed in this study are included in the published article and its Supplementary Materials. The raw sequence data were deposited in the NCBI SRA database with the BioProject accession number: PRJNA1436511.

## Supplementary Materials

The following supporting information can be downloaded at: https://www.mdpi.com/article/10.3390/antiox15050554/s1, Figure S1: Exogenous HRW represses ABA accumulation in postharvest NHCC leaves under dark and ABA induction. Figure S2: Exogenous hydrogen-rich water delays dark- and ABA-induced postharvest leaf senescence in CFC. Figure S3: Effects of exogenous HRW on ROS accumulation and antioxidant capacity in CFC leaves under dark and ABA stress conditions. Table S1: Primers used in this study.

## Abbreviations

The following abbreviations are used in this manuscript:

NHCC	Non-heading Chinese cabbage
CFC	Chinese flowering cabbage
HRW	Hydrogen-rich water
ABA	Abscisic acid
ROS	Reactive oxygen species
SAGs	Senescence-associated genes
CCGs	Chlorophyll catabolic genes
