# Supplementary material for: Hydrogen-Rich Water Suppresses Dark- and ABA-Induced Postharvest Senescence in Non-Heading Chinese Cabbage (Brassica rapa ssp. chinensis)
Source: Antioxidants (Basel). 2026 Apr 27;15(5):554. doi: 10.3390/antiox15050554 (PMC13203487; doi:10.3390/antiox15050554)
Supplement: Supplementary file 1 [file antioxidants-15-00554-s001.zip › antioxidants-4249510-supplementary.pdf]

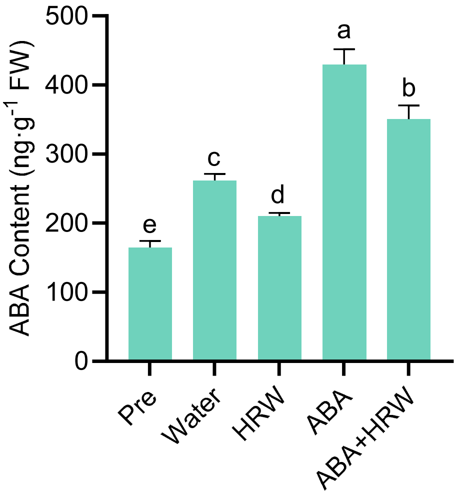

Figure S1. Exogenous HRW represses ABA accumulation in postharvest NHCC leaves under dark and ABA induction.

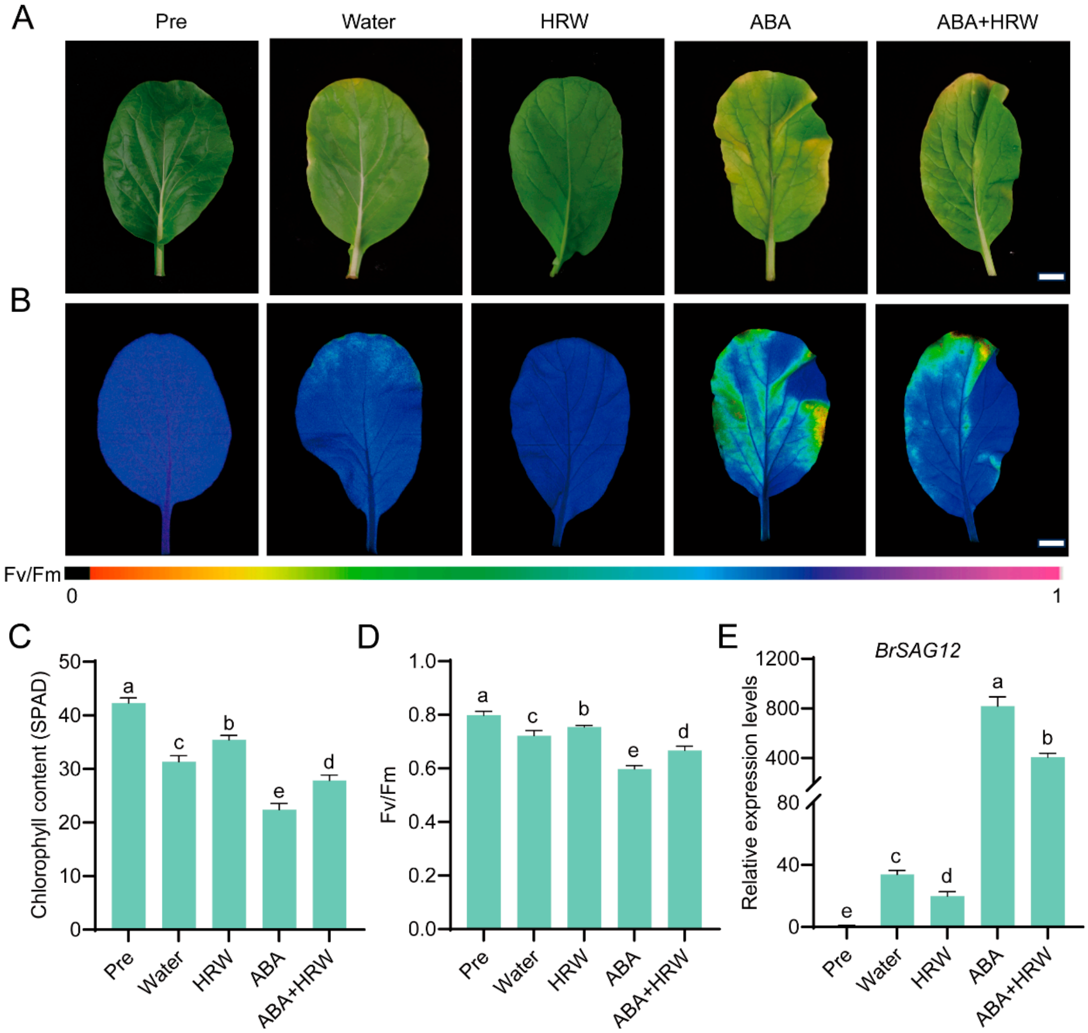

Figure S2. Exogenous hydrogen-rich water delays dark- and ABA-induced postharvest leaf senescence in CFC.

(A) Phenotype of Chinese flowering cabbage leaves after 3 days of dark storage at 22 °C. (B) Chlorophyll fluorescence images showing the maximum photochemical efficiency of photosystem

II (Fv/Fm). (C) Relative chlorophyll content (SPAD value). (D) Quantitative analysis of Fv/Fm. (E) Relative expression levels of the senescence marker gene *BrSAG12*. Bar= 1 cm. Data are means ± SD. Distinct letters denote significant differences (n = 3, $p < 0.05$, one-way ANOVA).

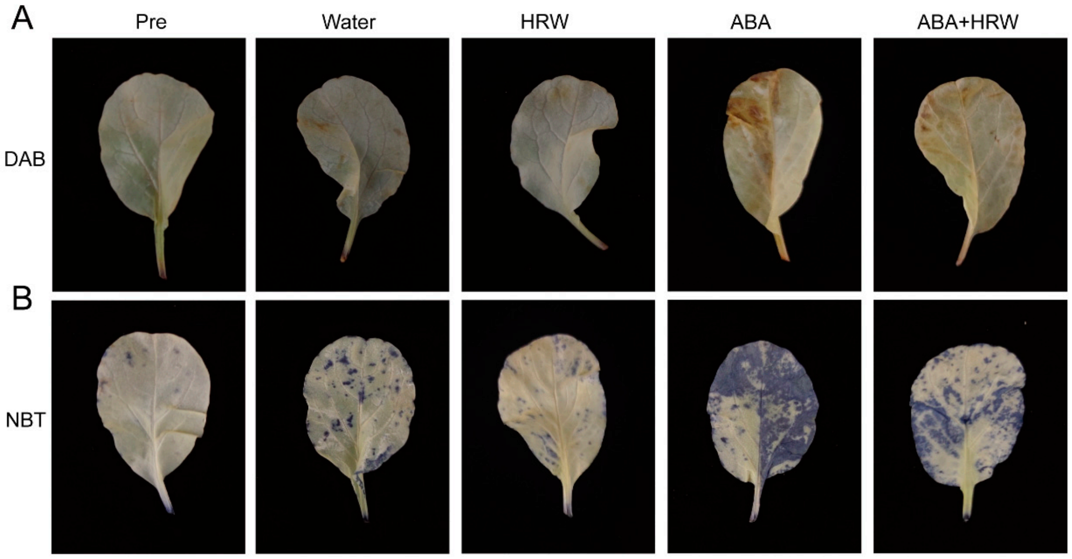

Figure S3. Effects of exogenous HRW on ROS accumulation and antioxidant capacity in CFC leaves under dark and ABA stress conditions.

(A) Histochemical localization of hydrogen peroxide (H_2O_2) via DAB staining. (B) Histochemical localization of superoxide anions ($O_2{\cdot}^-$) via NBT staining.

Supplemental Table S1. Primers used in this study

Primer Name	Primer Sequence (5'-3')	Note
BrNYC1-F	TGGAACCATGACCGGTGC	For qRT-PCR
BrNYC1-R	CCCTAGTCCCCTCGTGCT	For qRT-PCR
BrNOL-F	CAGAAAGCTTCGGCCTCTCA	For qRT-PCR
BrNOL-R	TCTCTCCTGAGATGCGAGCT	For qRT-PCR
BrHCAR-F	AAGTAGCGGTGATCGGCG	For qRT-PCR
BrHCAR-R	GGCTGAGCAGGACCTTGA	For qRT-PCR
BrNYE1-F	ATGCAGTTGTTGTTTCCCGC	For qRT-PCR
BrNYE1-R	GTCCCAGAGTAGCCACCAAC	For qRT-PCR
BrNYE2-F	AGGACTGCCTAAGTTTGCCC	For qRT-PCR
BrNYE2-R	AACCTCAGCACAGCGAATCA	For qRT-PCR
BrPPH1-F	CAGGGTTTGTCTCTCCCCAC	For qRT-PCR
BrPPH1-R	AACCCCCAAAACGGTTCACT	For qRT-PCR
BrPAO-F	CAGCTTCAGCGACACTCACC	For qRT-PCR
BrPAO-R	TCGCCGTGCTCTTCTTCGAT	For qRT-PCR
BrRCCR-F	ACGAACTGATGGCAGAGGTG	For qRT-PCR
BrRCCR-R	CCTAGCTCCTTCGCGATCTG	For qRT-PCR
BrSOD-F	GCACCCGAGGATGCTAATCG	For qRT-PCR
BrSOD-R	TACCACAAGCAACACGGCCT	For qRT-PCR
BrPOD-F	CTTAGTCGCGGCAGGGGAAT	For qRT-PCR
BrPOD-R	TCGACCTCGCAAACTGAGCA	For qRT-PCR
BrCAT-F	TCTGCAGCCTGTTGGACG	For qRT-PCR
BrCAT-R	GGACGCCAGGAACCACAA	For qRT-PCR
BrGPX1-F	AGTTCGGTGGCCAAGAGC	For qRT-PCR
BrGPX1-R	GTGCTGTGCTCGGTCCAT	For qRT-PCR
BrAPX1-F	CAGTCATGGTGCCAACAGCG	For qRT-PCR
BrAPX1-R	GGAAGTCGTCCTTCGTCCGT	For qRT-PCR
BrRbohB-F	CCTCCCGATGTGAAGCCACT	For qRT-PCR
BrRbohB-R	AGCTTCCCCGAAACAACCGA	For qRT-PCR
BrRbohC-F	TCTGAAACGCAACCGCCT	For qRT-PCR
BrRbohC-R	ACCGGCCTTCCTCGTACT	For qRT-PCR
BrRbohD-F	ACATCCGGACGCTTGGTG	For qRT-PCR
BrRbohD-R	TGGGAACGCAACGGAGTC	For qRT-PCR
BrRbohE-F	GGTTACGACGACGGTGTGGA	For qRT-PCR
BrRbohF-R	AGACGTGGCCTCCGACATTC	For qRT-PCR
BrABA1-F	ATGCTTGACTGGGTCCTTGG	For qRT-PCR
BrABA1-R	TCCGAAACGCTACACTTGCT	For qRT-PCR
BrABA2-F	TGGACAGCATGGGATACGTG	For qRT-PCR
BrABA2-R	ACCAACGCATCATCCGTTCT	For qRT-PCR

Primer Name	Primer Sequence (5'-3')	Note
BrABA3-F	AGACATGGAAACGGAGCTGG	For qRT-PCR
BrABA3-R	CAAGCTTCTCCACCTCCAGG	For qRT-PCR
BrCYP707A1-F	CCTTACGTGGGCGAGACTTT	For qRT-PCR
BrCYP707A1-R	TGAGACTTCGTCACCAGCAC	For qRT-PCR
BrCYP707A2-F	GCGCCAAAACCTTACACGTT	For qRT-PCR
BrCYP707A2-R	GTGGTGAGGTGGTGAAGGAG	For qRT-PCR
BrCYP707A3-F	CAACTTTTCCGGCGAGCAAA	For qRT-PCR
BrCYP707A3-R	AGCGACCATGTTCCTGACTG	For qRT-PCR
BrPYL5-F	TAAGATGATCCACGCGCCTC	For qRT-PCR
BrPYL5-R	CGTCCAAGATCTCGAGCCTC	For qRT-PCR
BrABI1-F	ATCATCGTCTTCGTCGTCGG	For qRT-PCR
BrABI1-R	CTCGGTATCGTGGAGACAGC	For qRT-PCR
BrABF3-F	TGGGGGAGATGACTCTGGAG	For qRT-PCR
BrABF3-R	ACGAGCTGAACCAAGACCTG	For qRT-PCR
